# Yellow Fever

**Published:** 1852-12

**Authors:** 


*Yellow Fever.*—This disease has prevailed to a considerable extent lately at Charleston, S. C. The daily number of deaths have averaged from ten to twenty. It has also prevailed at New Orleans and some other southern cities.

